# Comparison of Sociodemographic and Health-Related Characteristics of UK Biobank Participants With Those of the General Population

**DOI:** 10.1093/aje/kwx246

**Published:** 2017-06-21

**Authors:** Anna Fry, Thomas J Littlejohns, Cathie Sudlow, Nicola Doherty, Ligia Adamska, Tim Sprosen, Rory Collins, Naomi E Allen

**Keywords:** cancer, lifestyle, mortality, representativeness, sociodemographic characteristics, UK Biobank

## Abstract

The UK Biobank cohort is a population-based cohort of 500,000 participants recruited in the United Kingdom (UK) between 2006 and 2010. Approximately 9.2 million individuals aged 40–69 years who lived within 25 miles (40 km) of one of 22 assessment centers in England, Wales, and Scotland were invited to enter the cohort, and 5.5% participated in the baseline assessment. The representativeness of the UK Biobank cohort was investigated by comparing demographic characteristics between nonresponders and responders. Sociodemographic, physical, lifestyle, and health-related characteristics of the cohort were compared with nationally representative data sources. UK Biobank participants were more likely to be older, to be female, and to live in less socioeconomically deprived areas than nonparticipants. Compared with the general population, participants were less likely to be obese, to smoke, and to drink alcohol on a daily basis and had fewer self-reported health conditions. At age 70–74 years, rates of all-cause mortality and total cancer incidence were 46.2% and 11.8% lower, respectively, in men and 55.5% and 18.1% lower, respectively, in women than in the general population of the same age. UK Biobank is not representative of the sampling population; there is evidence of a “healthy volunteer” selection bias. Nonetheless, valid assessment of exposure-disease relationships may be widely generalizable and does not require participants to be representative of the population at large.

The UK Biobank Study is a large prospective cohort study, established primarily to investigate the genetic and lifestyle determinants of a wide range of diseases of middle and later life (1). This open-access resource involves 500,000 United Kingdom (UK) men and women who were aged 40–69 years when recruited throughout England, Wales, and Scotland between 2006 and 2010. Extensive questionnaire data, physical measurements, and biological samples were collected at recruitment, and there is ongoing enhanced data collection in large subsets of the cohort, including a repeat baseline assessment, genotyping, biochemical assays, Web-based questionnaires, physical activity monitoring, and multimodal imaging. All participants are followed up for health conditions through linkage to national electronic health-related data sets.

Our aim in the current study was to examine and quantify whether the UK Biobank cohort differed from the sampling frame with regard to a range of characteristics due to the “healthy volunteer effect” (2), whereby people who volunteer for research studies tend to be, on average, more health-conscious than nonparticipants (3). To investigate this, we compared the distributions of a range of sociodemographic, physical, lifestyle, and health-related characteristics between UK Biobank participants and 1) persons invited to join UK Biobank and 2) respondents to nationally representative surveys.

## METHODS

UK Biobank investigators sent postal invitations to 9,238,453 individuals registered with the UK's National Health Service who were aged 40–69 years and lived within approximately 25 miles (40 km) of one of 22 assessment centers located throughout England, Wales, and Scotland. The National Information Governance Board for Health and Social Care and the North West Multicentre Research Ethics Committee provided approval for UK Biobank to obtain the contact details of people within the eligible age range from local National Health Service Primary Care Trusts. UK Biobank also received approval to retain limited information on nonresponders. Overall, 503,317 participants consented to join the study cohort and visited an assessment center between 2006 and 2010, resulting in a participation rate of 5.45% (see Web Figure 1, available at https://academic.oup.com/aje, for a flow chart demonstrating responses to invitations).

Anonymized data on sex, month, and year of birth, Townsend deprivation index (an indicator of socioeconomic status), and geographic location are stored in the UK Biobank resource and were available for 8,761,869 of the 9,238,453 (94.8%) individuals sent an invitation letter, allowing us to compare the distributions of these characteristics between nonparticipating invitees and participants. The distributions of a range of sociodemographic, physical, lifestyle, and health-related characteristics of the UK Biobank cohort were also compared with publicly available summary data from nationally representative population-based surveys and the UK Census. We selected summary survey data that matched the UK Biobank cohort as closely as possible with regard to population demographic factors (i.e., both sexes and ages 40–69 years) and the period of data collection (2006–2010). Where certain characteristics from the national survey summary data were only available in prespecified aggregated age and sex subgroups, UK Biobank data were stratified into similar groups for comparative purposes. Formal statistical tests of the difference in characteristics between UK Biobank and national data were not performed because of the lack of variance measures required to test for differences between means, such as standard deviations, from the comparison populations.

The UK Census collects individual and household-level demographic data every 10 years for the whole UK population. Data on ethnicity were obtained from the 2001 and 2011 UK Census for England, Wales, and Scotland, as these reflected the census years falling before and immediately after the recruitment period (4, 5). Data on property ownership status were obtained from the 2001 UK Census for England and Wales only, since 2011 UK Census data on property ownership were not available for the appropriate age groups. Data on anthropometric measures, smoking status, alcohol consumption, and prevalences of self-reported health conditions were obtained from the Health Survey for England (HSE) for the years 2006, 2008, 2009, and 2010 (6–9). The HSE consists of an annual cross-sectional survey of a small (*n* = approximately 5,000–15,000), representative population of England through a 2-stage random probability sampling process, with information on different data items being collected in a different population each year (10, 11). Since 2003, the HSE has incorporated weighting to account for nonresponse bias (12). This includes different weights for nonresponding households, nonresponding individuals in responding households, and nonresponse at different stages of data collection. For a detailed description of the data collection methods used in UK Biobank and national surveys, see Web Table 1.

Age- and sex-specific data on all-cause mortality and cancer incidence rates for England were obtained from the Office for National Statistics for 2012, as this date represented the midpoint of the follow-up period for UK Biobank participants (13, 14). For all-cause mortality, follow-up time (person-years) in the UK Biobank cohort was calculated as the period ranging from age at recruitment to age at death or the date of complete follow-up (November 30, 2015), whichever came first; for cancer incidence rates, follow-up time was defined as the period ranging from age at recruitment to age at first cancer diagnosis, death, or the date of complete follow-up (September 30, 2014), whichever came first (among persons with no cancer at recruitment, based on cancer registry data). Cancer incidence rates were calculated for total cancer (excluding nonmelanoma skin cancer), defined using *International Classification of Diseases, Tenth Revision* (ICD-10), codes C00–C97 (excluding code C44), and common types—prostate (ICD-10 code C61), breast (ICD-10 code C50), colorectal (ICD-10 codes C18–C20), lung (ICD-10 codes C33–C34), endometrium (ICD-10 code C54), and kidney (ICD-10 code C64).

The UK Biobank Study received approval from the National Information Governance Board for Health and Social Care and the National Health Service North West Multicentre Research Ethics Committee.

## RESULTS

### Characteristics of UK Biobank participants versus nonparticipating invitees

**Figure 1.**
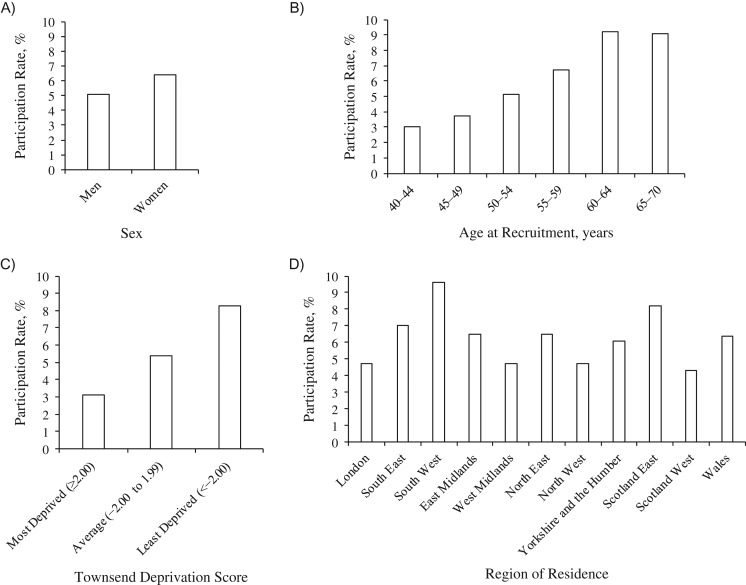
Rate of participation in the UK Biobank according to sex (A), age at recruitment (B), Townsend deprivation score (C), and region of residence (D), 2006–2010. For numerators and denominators, see Web Table 1. Participants were assigned a Townsend deprivation score corresponding to the output area of their residential postcode (most deprived: ≥2.00; average: −2.00 to 1.99; least deprived: <−2.00). UK, United Kingdom.

Of the 9,238,453 men and women invited to join UK Biobank, 503,317 (5.45%) consented and were recruited between 2006 and 2010. Overall, the participation rate was higher in women (participation rates were 6.4% and 5.1% in women and men, respectively) (Figure 1A), in older age groups (9% in those aged ≥60 years and 3% in those aged 40–44 years) (Figure 1B), and in less socioeconomically deprived areas (8.3% among persons from the least deprived areas and 3.1% among persons from the most deprived areas) (Figure 1C). Participation rates showed regional differences, being highest in South West England (9.6%) and East Scotland (8.2%) and lowest in West Scotland (4.3%), London, the West Midlands, and North West England (all 4.7%) (Figure 1D; also see Web Table 2 for further details).

### Characteristics of UK Biobank participants compared with national survey data

#### Sociodemographic factors

In the UK Biobank cohort, 94.6% of participants were of white ethnicity, which was similar to the national population of the same age range in the 2001 UK Census (94.5%) but somewhat higher than in the 2011 Census (91.3%; Table 1). UK Biobank participants were also more likely to own their property outright and were less likely to have a mortgage or loan, to share ownership, or to live in rental accommodations than the general population of the same age range (Table 2).

**Table 1.** Comparison of the Self-Reported Ethnic Origins of UK Biobank Participants (Recruited in 2006–2010) With Census Data for the Age Group 40–69 Years in England, Wales, and Scotland in 2001 and 2011^a^

Ethnicity^b^	UK Biobank (*n* = 499,877)		2001 UK Census (*n* = 20,198,307)		2011 UK Census (*n* = 23,146,612)	
	No. of Persons	%	No. of Persons	%	No. of Persons	%
White^c^	472,837	94.6	19,085,322	94.5	21,133,317	91.3
Black or black British^d^	8,066	1.6	302,073	1.5	565,777	2.4
Mixed^e^	2,958	0.6	82,389	0.4	191,085	0.8
Indian	5,951	1.2	325,651	1.6	442,338	1.9
Pakistani	1,837	0.4	147,695	0.7	239,166	1.0
Bangladeshi	236	0.0	46,220	0.2	75,919	0.3
Chinese	1,574	0.3	70,572	0.3	109,412	0.5
Other Asian	1,858	0.4	73,917	0.4	240,324	1.0
Other ethnic group	4,560	0.9	64,468	0.3	149,274	0.6

Abbreviation: UK, United Kingdom.

^a^ See references 4 and 5 for further information about census data.

^b^ Excludes 2,778 UK Biobank participants aged 40–69 years who were missing data on ethnicity or responded “prefer not to answer” or “do not know.”

^c^ Included white British, white Irish, and other white background.

^d^ Included Caribbean, African, and other black background.

^e^ Included white and black Caribbean, white and black African, white and Asian, and other mixed ethnic background.

**Table 2.** Comparison of the Property Ownership Status of UK Biobank Participants (Recruited in 2006–2010) With Census Data for the Age Group 50–64 Years in England and Wales in 2001^a^

Property Ownership Status^b^	UK Biobank (*n* = 284,400)		2001 UK Census (*n* = 9,098,700)	
	No. of Persons	%	No. of Persons	%
Owned outright	161,318	56.7	3,690,996	40.6
Owned with mortgage or loan	96,427	33.9	3,599,560	39.6
Shared ownership	682	0.2	33,971	0.4
Rented from council (local authority), housing association, or registered social landlord	16,407	5.8	1,187,422	13.1
Rented from private landlord/letting agency	7,514	2.6	418,900	4.6
Living in accommodation rent-free	2,052	0.7	117,344	1.3
Living in a communal establishment^c^	N/A	N/A	49,877	0.5

Abbreviations: N/A, not available; UK, United Kingdom.

^a^ See the 2001 UK Census aggregate data set (4) for further information about census data.

^b^ Excludes 4,313 UK Biobank participants aged 50–64 years who were missing data on property ownership status or who responded “none of the above” or “prefer not to answer.”

^c^ Category not included in the UK Biobank questionnaire.

#### Physical characteristics

UK Biobank participants were, on average, taller and leaner and had a smaller waist circumference than the general population, based on the HSE 2008 (Table 3). For example, mean body mass index (defined as weight (kg)/height (m)^2^) in UK Biobank men and women aged 55–64 years was 27.9 and 27.3, respectively, as compared with 28.5 and 28.0 in the general population, based on data from the HSE 2008. UK Biobank men and women were also less likely to be obese (defined as body mass index ≥30) across all age groups examined in comparison with the general population. For example, for men aged 45–54 years, the prevalence of obesity was 25.6% in UK Biobank and 31.5% in the general population, with corresponding values of 23.0% and 32.2%, respectively, for women (Web Table 3).

**Table 3.** Comparison of Mean Levels of Anthropometric Measures, by Age and Sex, for UK Biobank Participants (Recruited in 2006–2010) With Data From the Health Survey for England 2008^a,b^

Sex and Anthropometric Measure^c^	Age 45–54 Years				Age 55–64 Years			
	UK Biobank		HSE		UK Biobank		HSE	
	No. of Persons	Mean (SD)	No. of Persons	Mean^d^	No. of Persons	Mean (SD)	No. of Persons	Mean^d^
Men								
BMI^e^	61,860	27.8 (4.4)	1,059	28.1	94,776	27.9 (4.3)	968	28.5
Weight, kg	61,929	86.9 (15.1)	1,079	86.4	94,875	86.0 (14.3)	980	86.7
Height, cm	61,919	176.5 (6.9)	1,076	175.1	94,901	175.4 (6.7)	981	174.0
WC, cm^f^	62,010	96.1 (11.5)	845	100.3	95,031	97.7 (11.4)	755	102.9
Women								
BMI	79,714	26.9 (5.4)	1,057	27.7	116,303	27.3 (5.1)	985	28.0
Weight, kg	79,738	71.8 (14.8)	1,067	72.8	116,344	71.6 (13.8)	995	72.3
Height, cm	79,792	163.4 (6.3)	1,097	162.0	116,429	162.0 (6.2)	1,016	160.5
WC, cm^f^	79,809	83.6 (12.8)	850	89.3	116,471	85.5 (12.5)	784	91.6

Abbreviations: BMI, body mass index; HSE, Health Survey for England; SD, standard deviation; UK, United Kingdom; WC waist circumference.

^a^ See the HSE 2010 (9) for further information about HSE data.

^b^ HSE data were weighted for nonresponse bias.

^c^ Excludes UK Biobank participants aged 45–64 years with missing data for BMI (*n* = 2,158), weight (*n* = 1,925), height (*n* = 1,770), or WC (*n* = 1,482).

^d^ SDs were not available from the HSE.

^e^ Weight (kg)/height (m)^2^.

^f^ Additionally excludes 8 UK Biobank participants aged 45–64 years for whom WC values outside the range of 50–180 cm were obtained.

#### Lifestyle characteristics

**Figure 2.**
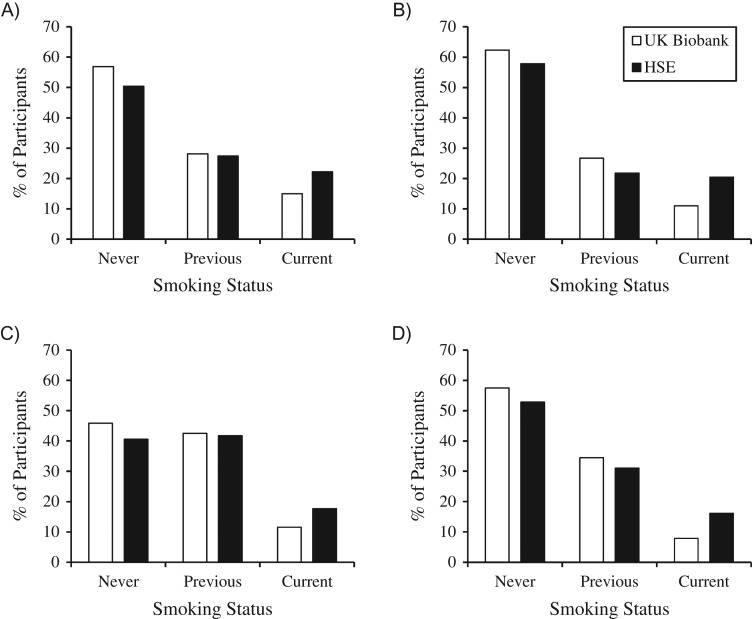
Comparison of smoking status in UK Biobank participants (recruited in 2006–2010) with data from the Health Survey for England (HSE) 2008 for men aged 45–54 years (A), women aged 45–54 years (B), men aged 55–64 years (C), and women aged 55–64 years (D). HSE estimates were weighted for nonresponse bias. The graph excludes 1,899 UK Biobank participants aged 45–64 years who had missing data on smoking status or responded “prefer not to answer.” Numbers of participants: A) UK Biobank, *n* = 62,004; HSE, *n* = 1,206; B) UK Biobank, *n* = 79,755; HSE, *n* = 1,233; C) UK Biobank, *n* = 94,907; HSE, *n* = 1,085; D) UK Biobank, *n* = 116,246; HSE, *n* = 1,123. See HSE 2010 (9) for further information about HSE data. UK, United Kingdom.

UK Biobank men and women were less likely to be current smokers than the general population across all age groups, based on data from the HSE 2008 (Figure 2). For example, for men aged 45–54 years, the prevalence of current smoking was 15% in UK Biobank and 22% in the general population; the corresponding values for women were 11% and 20%, respectively. However, younger smokers (aged 45–54 years) in UK Biobank smoked more heavily (≥20 cigarettes/day) than those in the general population (46% and 41%, respectively, for men; 32% and 28%, respectively, for women). This difference persisted for older women aged 55–64 years (31% and 23% in UK Biobank and the general population, respectively) but not for older men (47% and 49%, respectively; Web Figure 2). UK Biobank participants were also less likely to be never drinkers but were less likely to drink alcohol every day than the general population included in the HSE 2008 (Table 4).

**Table 4.** Comparison of Data (%) on the Frequency of Alcohol Consumption, by Age and Sex, Among UK Biobank Participants (Recruited in 2006–2010) With Data From the Health Survey for England 2008^a,b^

Alcohol Consumption^c^	Men				Women			
	Age 45–54 Years		Age 55–64 Years		Age 45–54 Years		Age 55–64 Years	
	UK Biobank (*n* = 62,082)	HSE (*n* = 1,204)	UK Biobank (*n* = 95,207)	HSE (*n* = 1,085)	UK Biobank (*n* = 79,904)	HSE (*n* = 1,232)	UK Biobank (*n* = 116,605)	HSE (*n* = 1,123)
Daily^d^	21.2	24	28.3	30	14.5	16	17.6	18
3–4 days/week	26.8	21	26.9	15	21.9	16	20.9	15
1–2 days/week	28.2	29	24.2	26	27.6	26	24.9	23
1–3 times/month	10.0	10	8.0	9	13.9	12	12.2	11
Special occasions^e^	7.4	9	6.8	11	13.8	16	15.0	21
Never^f^	6.6	8	5.8	9	8.3	12	9.5	12

Abbreviations: HSE, Health Survey for England; UK, United Kingdom.

^a^ See the HSE 2010 (9) for further information about HSE data.

^b^ HSE estimates were weighted for nonresponse bias.

^c^ Excludes 1,013 UK Biobank participants aged 45–64 years who were missing data for alcohol intake or responded “prefer not to answer.”

^d^ The HSE categories “almost every day” and “5 or 6 days a week” were defined as “daily.”

^e^ The HSE categories “once every couple of months” and “once or twice in the past year” were defined as “special occasions.”

^f^ The HSE category “not at all in the last 12 months/nondrinker” was defined as “never.”

#### Self-reported health conditions

UK Biobank participants had a lower prevalence of self-reported health conditions, including cardiovascular disease, stroke, hypertension, diabetes, chronic kidney disease, and respiratory disease, than the general population, as obtained from various HSEs performed in 2006, 2009, and 2010 (Table 5). For example, among men aged 45–54 years, the prevalence of self-reported cardiovascular disease was 4.6% in UK Biobank participants and 10.9% in the general population, and among women aged 45–54 years the prevalences were 2.4% and 10.3%, respectively.

**Table 5.** Comparison of the Prevalence (%) of Self-Reported Health Conditions, by Age and Sex, in UK Biobank Participants (Recruited in 2006–2010) With Data From the Health Survey for England 2006, 2009, or 2010^a,b,c^

Self-Reported Disease	Men				Women			
	Age 45–54 Years		Age 55–64 Years		Age 45–54 Years		Age 55–64 Years	
	UK Biobank	HSE	UK Biobank	HSE	UK Biobank	HSE	UK Biobank	HSE
Cardiovascular disease^d^	4.6	10.9	11.5	18.5	2.4	10.3	5.0	15.2
Ischemic heart disease^e^	2.8	3.6	7.9	10.6	0.9	1.3	2.6	3.5
Stroke	0.8	1.2	1.9	3.0	0.6	0.9	1.0	2.3
Angina	1.8	2.4	5.3	8.0	0.7	1.2	2.1	3.2
Myocardial infarction	1.7	2.1	4.5	6.3	0.3	0.7	0.9	1.6
Abnormal heart rhythm	1.5	5.7	3.1	6.3	1.4	5.7	2.2	7.3
Hypertension^f^	21.2	27	34.4	39	15.4	16	27.4	29
Diabetes	4.5	8.1	7.8	10.5	2.4	3.5	6.3	8.0
Chronic kidney disease	0.2	1.1	0.3	1.5	0.2	1.2	0.2	1.9
Asthma^f^	11.7	12	9.9	13	13.0	16	11.8	15
COPD^f^	0.1	1	0.4	3	0.1	0	0.4	2

Abbreviations: COPD, chronic obstructive pulmonary disease; HSE, Health Survey for England; UK, United Kingdom.

^a^ See references 8, 10, and 11 for further information about HSE data.

^b^ HSE estimates were weighted for nonresponse bias.

^c^ HSE 2006 data were used for cardiovascular disease, ischemic heart disease, stroke, angina, myocardial infarction, and abnormal heart rhythm (*n* = 1,123, *n* = 1,015, *n* = 1,141, and *n* = 1,050, respectively). HSE 2009 estimates were used for hypertension (*n* = 274, *n* = 244, *n* = 280, and *n* = 253, respectively) and diabetes (*n* = 391, *n* = 345, *n* = 398, and *n* = 358, respectively). HSE 2010 estimates were used for asthma (*n* = 720, *n* = 608, *n* = 730, and *n* = 630, respectively) and COPD (*n* = 720, *n* = 608, *n* = 730, and *n* = 631, respectively). Both 2009 and 2010 estimates (*n* = 1,112, *n* = 1,128, *n* = 953, and *n* = 989, respectively) were used for chronic kidney disease.

^d^ Cardiovascular disease included angina, heart attack, stroke, heart murmur, and irregular heart rhythm.

^e^ Ischemic heart disease included heart attack or angina.

^f^ HSE estimates were available only to the nearest integer.

#### All-cause mortality and cancer incidence rates

**Figure 3.**
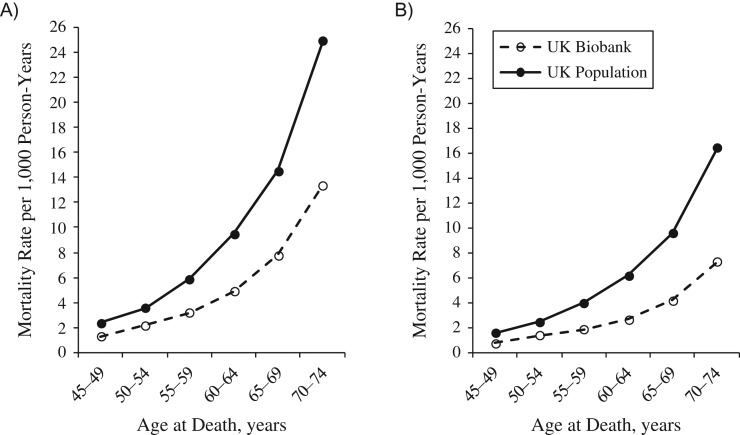
Comparison of mortality rates per 1,000 person-years, by age at death, for UK Biobank participants (recruited in 2006–2010) and the population of England and Wales in 2012 (data from the Office for National Statistics) for men (A) and women (B). Total number of deaths in UK Biobank participants aged 45–74 years: men, 8,291; women, 5,380. See United Kingdom Office for National Statistics (13) for further information about death registration data. UK, United Kingdom.

**Figure 4.**
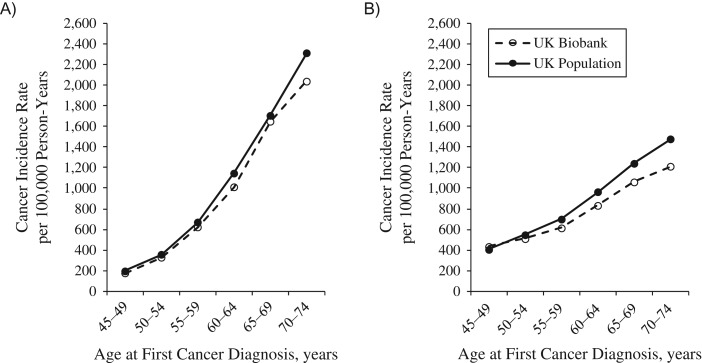
Comparison of incidence rates for all cancers (excluding nonmelanoma skin cancer) per 100,000 person-years, by age at cancer diagnosis, for UK Biobank participants (recruited in 2006–2010) and the population of England in 2012 (data from the Office for National Statistics) for men (A) and women (B). Total number of all incident cancers (excluding nonmelanoma skin cancer) in UK Biobank participants aged 45–74 years: men, 11,436; women, 10,592. See United Kingdom Office for National Statistics (14) for further information about cancer registration data. UK, United Kingdom.

UK Biobank participants were followed up for mean durations of 6.77 (standard deviation, 1.01) years and 5.53 (standard deviation, 1.10) years for all-cause mortality and incident cancer, respectively. Compared with national death rates among persons aged 70–74 years, all-cause mortality in UK Biobank participants was 46.2% lower in men and 55.5% lower in women (Figure 3A and 3B; also see Web Table 4 for further details of age-specific mortality rates). The total cancer incidence rate was also lower than in the general population, being 11.8% and 18.1% lower at ages 70–74 years in men and women, respectively (Figure 4A and 4B; also see Web Table 5 for further details of age-specific cancer incidence rates). A similar pattern was observed for cancers of the colorectum, kidney, and endometrium (Web Figure 3). Lung cancer incidence rates in UK Biobank were markedly lower for both men and women, while rates of female breast cancer were similar to the national average, with the exception of women aged 45–49 years, in whom the rate was higher in the UK Biobank cohort. In contrast, prostate cancer incidence was higher in UK Biobank compared with national rates across all age groups examined.

## DISCUSSION

The rate of participation in the UK Biobank Study was higher among women, older age groups, and persons living in less socioeconomically deprived areas. UK Biobank participants also differed with regard to several lifestyle and health-related characteristics when compared with the general population of the same age. For example, men aged 45–54 years were less likely to be obese (25.6% in UK Biobank vs. 31.5% in the general population) and less likely to be current smokers (15% vs. 22%), with similar findings being observed for women and older age groups. Furthermore, compared with the general population, UK Biobank participants were less likely to drink alcohol on a daily basis and had fewer self-reported health conditions. Linkage of UK Biobank participants with their health records during an average of 6–7 years of follow-up also showed lower rates of all-cause mortality and total cancer incidence than in the general population of the same age.

These findings are consistent with the well-established “healthy volunteer” effect, which has been demonstrated in other volunteer-based cohort studies (15–17). Other prospective studies have also found lower rates of all-cause mortality and incident cancer in comparison with national rates (18–21). The only examined health condition that had a higher incidence rate in UK Biobank than in the general population was prostate cancer, which might reflect higher rates of voluntary prostate-specific antigen testing (and subsequent prostate cancer diagnosis) among health-conscious men. In contrast, lung cancer incidence rates were markedly lower in UK Biobank across all age and sex groups, almost certainly caused by the lower prevalence of smoking compared with the general population.

Because UK Biobank participants are, on average, more health-conscious than the general population, this cohort is not the best for estimation of generalizable prevalence or incidence rates of disease (although some health-related characteristics of the UK Biobank cohort, such as the prevalence of self-reported pain, have previously been shown to be similar to those of the national population (22)). In order for a cohort study to produce generalizable associations of exposures with disease, it is important that sufficiently large numbers of individuals with different levels of exposures be investigated with high internal validity (23–26). Indeed, if one were interested in investigating the association of ethnicity with subsequent disease risk, the most appropriate study design would be to recruit a large number of people from different ethnic backgrounds rather than have a representative, largely white population. Because UK Biobank is primarily designed for investigating exposure-disease associations, the lack of representativeness should not be regarded as a limitation (27, 28). As with all observational studies, it is incumbent upon researchers to acknowledge potential sources of bias that might affect the generalizability of exposure-disease associations on a case-by-case basis, such as residual confounding, reverse causation, and self-selection bias (24, 29). Although the UK Biobank Study is still in the early stages as a prospective study, initial publications have shown expected associations of cardiometabolic morbidity, self-reported health, and smoking with mortality risk (30, 31).

This study provides an overview of the representativeness of the UK Biobank cohort with regard to a variety of key characteristics in comparison with the general UK population using data from nationally representative surveys. We expect that these findings will be used by researchers to inform the interpretation of results or, in some instances, to help generate weighted results (e.g., in order to estimate nationally representative disease rates). We were able to compare participation rates for key sociodemographic characteristics (such as age, sex, socioeconomic status, and geographic location) due to the availability of such data for the total sampling frame. The availability of follow-up health data enabled us to compare death and cancer incidence rates with age- and sex-specific national rates, and the large size of the cohort meant that sufficient numbers of cases had accrued to investigate common cancer types. All UK Biobank participants are flagged by national death and cancer registries, and loss to follow-up due to emigration has been minimal (0.3% of the cohort). Further follow-up is required to determine whether this “healthy volunteer effect” attenuates over time (owing to the development of chronic disease as the cohort ages), a phenomenon which has been observed in previous studies (18, 20, 32).

One limitation of our study is that the national survey data (available from the UK Census and the HSE) were presented in prespecified age groups, thereby restricting the comparisons that could be performed. For the majority of characteristics, comparable national survey data were available only for England, although only 11% of participants were recruited in Wales and Scotland and the distributions of most characteristics were similar across the 3 countries. It is also possible that differences in the wording of questions, answer choices, and data collection methods might have influenced the comparability of certain characteristics between the national surveys and the UK Biobank cohort. For example, the HSE consisted primarily of a verbal interview that enabled the interviewer to probe the participant for further information, whereas data on all of the characteristics of UK Biobank participants presented here were collected via a touchscreen questionnaire, with the exception of information on self-reported health conditions, which was collected through a verbal interview with a trained nurse.

In conclusion, the UK Biobank cohort is not representative of the general population with regard to a number of sociodemographic, physical, lifestyle, and health-related characteristics. UK Biobank participants generally live in less socioeconomically deprived areas; are less likely to be obese, to smoke, and to drink alcohol on a daily basis; and have fewer self-reported health conditions. All-cause mortality is approximately half that of the UK population as a whole, and total cancer incidence rates are approximately 10%–20% lower. Although UK Biobank is not suitable for deriving generalizable disease prevalence and incidence rates, its large size and heterogeneity of exposure measures provide valid scientific inferences of associations between exposures and health conditions that are generalizable to other populations.

## Supplementary Material

Web Material

## References

[1] Sudlow C, Gallacher J, Allen N, et al UK Biobank: an open access resource for identifying the causes of a wide range of complex diseases of middle and old age. PLoS Med. 2015;12(3):e1001779. 25826379 10.1371/journal.pmed.1001779 PMC4380465

[2] Delgado-Rodriguez M, Llorca J Bias. J Epidemiol Community Health. 2004;58(8):635–641. 15252064 10.1136/jech.2003.008466 PMC1732856

[3] Manolio TA, Weis BK, Cowie CC, et al New models for large prospective studies: is there a better way? Am J Epidemiol. 2012;175(9):859–866. 22411865 10.1093/aje/kwr453 PMC3339313

[4] Office for National Statistics; General Register Office for Scotland; Northern Ireland Statistics and Research Agency 2001 Census aggregate [data set]. https://discover.ukdataservice.ac.uk/doi/2001-census-aggregate Published 2005. Updated June 2016. Accessed September 1, 2016.

[5] Office for National Statistics; National Records of Scotland; Northern Ireland Statistics and Research Agency 2011 Census aggregate [data set]. https://discover.ukdataservice.ac.uk/doi/2011-census-aggregate Published 2005. Accessed September 1, 2016.

[6] United Kingdom National Health Service Health Survey for England–2006: CVD and risk factors for adults, obesity and risk factors for children. http://content.digital.nhs.uk/catalogue/PUB01213 Published January 31, 2008. Accessed December 22, 2015.

[7] United Kingdom National Health Service. Health Survey for England–2008: physical activity and fitness. http://content.digital.nhs.uk/catalogue/PUB00430 Published December 17, 2009. Accessed December 22, 2015.

[8] United Kingdom National Health Service. Health Survey for England–2009: health and lifestyles. http://content.digital.nhs.uk/catalogue/PUB00414 Published December 16, 2010. Accessed December 22, 2015.

[9] United Kingdom National Health Service. Health Survey for England–2010: respiratory health. http://content.digital.nhs.uk/catalogue/PUB03023 Published December 15, 2011. Accessed December 22, 2015.

[10] Mindell J, Aresu M, Bécares L, et al Representativeness of participants in a cross-sectional health survey by time of day and day of week of data collection. Eur J Public Health. 2012;22(3):364–369. 21965544 10.1093/eurpub/ckr093

[11] Mindell J, Biddulph JP, Hirani V, et al Cohort profile: the Health Survey for England. Int J Epidemiol. 2012;41(6):1585–1593. 22253315 10.1093/ije/dyr199

[12] National Centre for Social Research *Health Survey for England 2003. Volume 3. Methodology and Documentation* London, United Kingdom: Department of Health; 2004 http://webarchive.nationalarchives.gov.uk/20121206162012/http://www.dh.gov.uk/prod_consum_dh/groups/dh_digitalassets/@dh/@en/documents/digitalasset/dh_4098912.pdf. Published December 17, 2004. Accessed December 22, 2015.

[13] United Kingdom Office for National Statistics Death registration summary tables—England and Wales: 2012. http://www.ons.gov.uk/ Published July 10, 2013. Accessed May 1, 2016.

[14] United Kingdom Office for National Statistics Cancer statistics registrations, England: 2012. http://www.ons.gov.uk/ Published June 19, 2014. Accessed May 1, 2016.

[15] Andreeva VA, Salanave B, Castetbon K, et al Comparison of the sociodemographic characteristics of the large NutriNet-Santé e-cohort with French Census data: the issue of volunteer bias revisited. J Epidemiol Community Health. 2015;69(9):893–898. 25832451 10.1136/jech-2014-205263

[16] Mishra GD, Hockey R, Powers J, et al Recruitment via the Internet and social networking sites: the 1989–1995 cohort of the Australian Longitudinal Study on Women's Health. J Med Internet Res. 2014;16(12):e279. 25514159 10.2196/jmir.3788 PMC4275491

[17] Brown WJ, Bryson L, Byles JE, et al Women's Health Australia: recruitment for a national longitudinal cohort study. Women Health. 1999;28(1):23–40. 10.1300/j013v28n01_03 10022055

[18] Struijk E, May A, Beulens J, et al Mortality and cancer incidence in the EPIC-NL cohort: impact of the healthy volunteer effect. Eur J Public Health. 2015;25(1):144–149. 24736167 10.1093/eurpub/cku045

[19] Otto SJ, Schroder FH, de Koning HJ Low all-cause mortality in the volunteer-based Rotterdam section of the European randomised study of screening for prostate cancer: self-selection bias? J Med Screen. 2004;11(2):89–92. 15153324 10.1258/096914104774061074

[20] Pinsky PF, Miller A, Kramer BS, et al Evidence of a healthy volunteer effect in the Prostate, Lung, Colorectal, and Ovarian Cancer Screening Trial. Am J Epidemiol. 2007;165(8):874–881. 17244633 10.1093/aje/kwk075

[21] Lindsted KD, Fraser GE, Steinkohl M, et al Healthy volunteer effect in a cohort study: temporal resolution in the Adventist Health Study. J Clin Epidemiol. 1996;49(7):783–790. 8691229 10.1016/0895-4356(96)00009-1

[22] Macfarlane GJ, Beasley M, Smith BH, et al Can large surveys conducted on highly selected populations provide valid information on the epidemiology of common health conditions? An analysis of UK Biobank data on musculoskeletal pain. Br J Pain. 2015;9(4):203–212. 26526341 10.1177/2049463715569806 PMC4616980

[23] Rothman K, Gallacher J, Hatch E Why representativeness should be avoided. Int J Epidemiol. 2013;42(4):1012–1014. 24062287 10.1093/ije/dys223 PMC3888189

[24] Ebrahim S, Davey Smith G Commentary: should we always deliberately be non-representative? Int J Epidemiol. 2013;42(4):1022–1026. 24062291 10.1093/ije/dyt105

[25] Elwood J Commentary: on representativeness. Int J Epidemiol. 2013;42(4):1014–1015. 24062288 10.1093/ije/dyt101

[26] Richiardi L, Pizzi C, Pearce N Commentary: representativeness is usually not necessary and often should be avoided. Int J Epidemiol. 2013;42(4):1018–1022. 24062290 10.1093/ije/dyt103

[27] Allen N, Sudlow C, Downey P, et al UK Biobank: current status and what it means for epidemiology. Health Policy Technol. 2012;1(3):123–126.

[28] Collins R What makes UK Biobank special? Lancet. 2012;379(9822):1173–1174. 22463865 10.1016/S0140-6736(12)60404-8

[29] Hernán MA, Hernández-Díaz S, Robins JM A structural approach to selection bias. Epidemiology. 2004;15(5):615–625. 15308962 10.1097/01.ede.0000135174.63482.43

[30] Emerging Risk Factors Collaboration, Di Angelantonio E, Kaptoge S, et al Association of cardiometabolic multimorbidity with mortality. JAMA. 2015;314(1):52–60. 26151266 10.1001/jama.2015.7008 PMC4664176

[31] Ganna A, Ingelsson E 5 year mortality predictors in 498 103 UK Biobank participants: a prospective population-based study. Lancet. 2015;386(9993):533–540. 26049253 10.1016/S0140-6736(15)60175-1

[32] Burnell M, Gentry-Maharaj A, Ryan A, et al Impact on mortality and cancer incidence rates of using random invitation from population registers for recruitment to trials. Trials. 2011;12:61. 21362184 10.1186/1745-6215-12-61 PMC3058013

