# Supplementary material for: Comparison of Sociodemographic and Health-Related Characteristics of UK Biobank Participants With Those of the General Population
Source: Am J Epidemiol. 2017 Jun 21;186(9):1026–34. doi: 10.1093/aje/kwx246 (PMC5860371; doi:10.1093/aje/kwx246)
Supplement: Web Material [file kwx246littlejohnswebmaterialfinal.pdf]

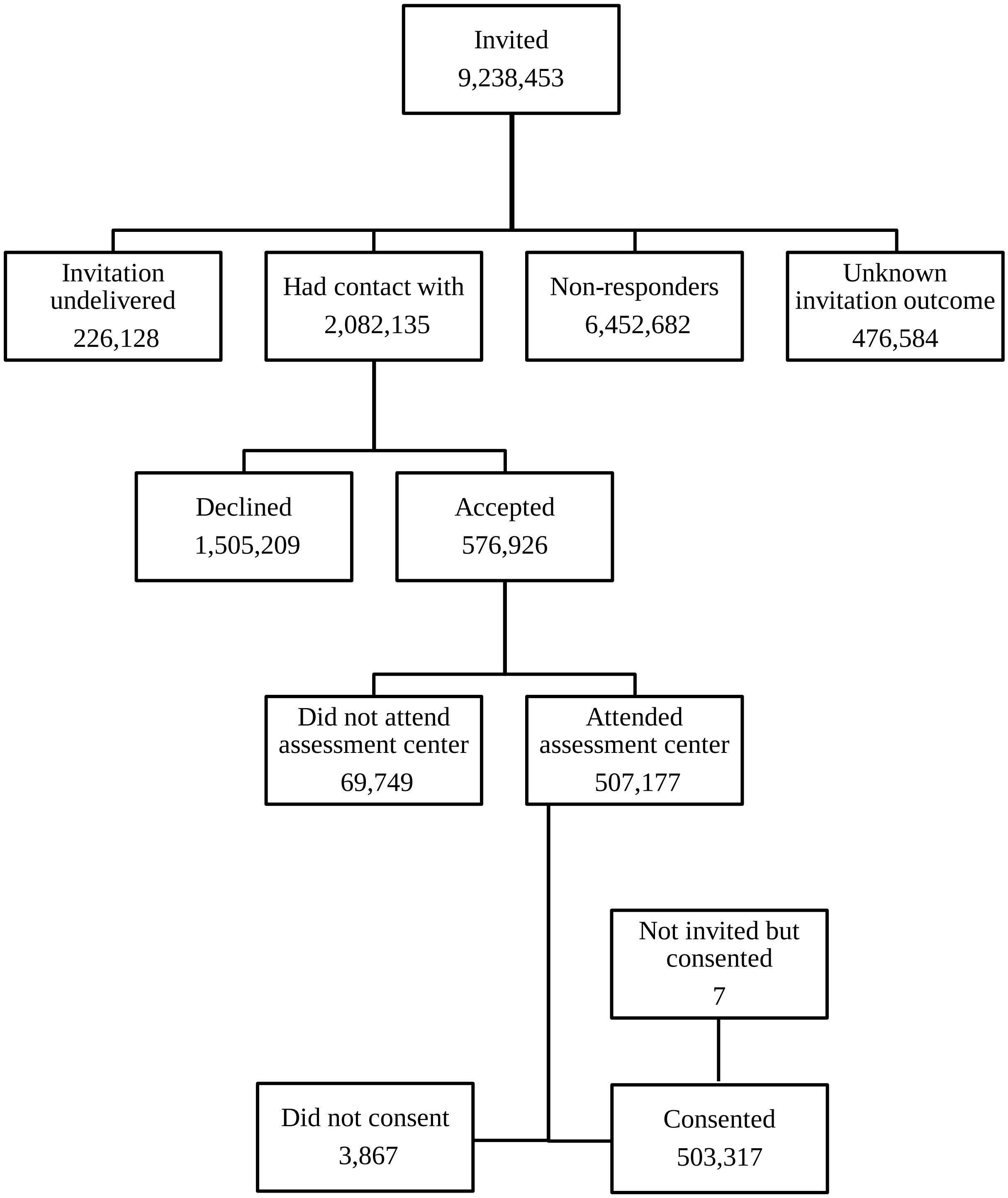

Web Figure 1. Diagram demonstrating flow of participants from invitation to recruitment. Seven participants were not invited but nonetheless attended an assessment center and were consented to join UK Biobank.

Web Table 1. Details of Collection Methods for Each Characteristic as Collected From Nationally Representative Surveys and UK Biobank

Characteristic	Survey	Collection Method in Survey	Collection Method in UK Biobank
Ethnicity	UK Census for England, Wales and Scotland 2001 and 2011	Paper-based (2001 and 2011) or online (2011) questionnaire<br><br>2001 England and Wales<br><br>'*What is your ethnic group?*'—multiple choice response; either 1) White OR 2) Mixed OR 3) Asian or Asian British* OR 4) Black or Black British OR 5) Chinese or other ethnic group<br><br>*Subgroups include Indian, Pakistani, Bangladeshi or any other Asian background<br><br>2001 Scotland<br><br>'*What is your ethnic group?*'—multiple choice response; either 1) White OR 2) Mixed OR 3) Asian, Asian Scottish or Asian British* OR 4) Black, Black Scottish or Black British OR 5) Other ethnic background<br><br>*Subgroups include Indian, Pakistani, Bangladeshi, Chinese or any other Asian background<br><br>2011 England and Wales	Touchscreen questionnaire<br><br>Ethnicity was derived from two questions:<br><br>'*What is your ethnic group?*'—multiple choice response; either 1) White OR 2) Mixed OR 3) Asian or Asian British OR 4) Black or Black British OR 5) Chinese OR 6) Other ethnic group OR 7) Do not know OR 8) Prefer not to answer.<br><br>'*What is your ethnic background?*'—multiple choice response; either 1) Indian OR 2) Pakistani OR 3) Bangladeshi OR 4) Any other Asian background

		‘*What is your ethnic group?*’—multiple choice response; either 1) White OR 2) Mixed/multiple ethnic groups OR 3) Asian/ Asian British* OR 4) Black/African/Caribbean/Black British OR 5) Other ethnic group<br><br>*Subgroups include Indian, Pakistani, Bangladeshi, Chinese or any other Asian background<br><br>2011 Scotland<br><br>‘*What is your ethnic group?*’—multiple choice response; either 1) White OR 2) Mixed or multiple ethnic groups OR 3) Asian, Asian Scottish or Asian British* OR 4) African OR 5) Caribbean or Black OR 6) Other ethnic group<br><br>*Subgroups include Indian, Pakistani, Bangladeshi, Chinese or other	
Tenure status	UK Census for England and Wales 2001	Paper-based questionnaire<br><br>Tenure status derived from two questions<br><br>‘*Does your household own or rent the accommodation?*’—multiple choice response; either 1) Owns outright OR 2) Owns with a mortgage or loan OR 3) Pays part rent and part mortgage (shared ownership) OR 4) Rents OR 5) Lives here rent free	Touchscreen questionnaire<br><br>‘*Do you own or rent the accommodation that you live in?*’—multiple choice response; either 1) Own outright (by you or someone in your household) OR 2) Own with a mortgage OR 3) Rent—from local authority, local council, housing association OR 4) Rent—from private landlord or letting agency OR 5) Pay part rent and part mortgage (shared ownership)

		'*Who is your landlord?*'—multiple choice response; either 1) Council (Local Authority) OR 2) Housing Association, Housing Co-operative, Charitable Trust or registered landlord OR 3) Private landlord or letting agency OR 4) Employer of a household member OR 5) Relative or friend of a household member OR 6) Other<br><br>Individuals living in communal establishments (i.e. care homes, hotels, military barracks etc.) were identified using a Communal Establishment paper form which was completed and returned by the manager or person in charge	OR 6) Live in accommodation rent free OR 7) None of the above OR 8) Prefer not to answer
BMI	HSE 2008[a]	BMI derived using weight (kg) and height (m) using the following formula; BMI = kg/m^2	BMI was derived using weight (kg) and height (m) using the following formula; BMI = kg/m^2
Weight	HSE 2008[a]	Weight in kg was measured using Soehnle, Seca or Tanita electronic scales which accurately measures weight to within 0.1 kg<br><br>Participants were asked to remove their shoes and items of heavy clothing<br><br>Participants who were pregnant, unable to stand or unsteady on their feet were not weighed	Weight (kg) was measured using a Tanita BC-418 MA body composition analyser which accurately measures weight to within 0.1 kg. Participants were asked to remove their shoes and items of heavy clothing.
Standing height	HSE 2008[a]	Standing height in cm was measured using a portable stadiometer<br><br>Participants who were unable to stand or unsteady on their feet were not measured<br><br>Participants were asked to remove their shoes	Standing height (cm) was measured using a Seca 202 height measure. Participants were asked to remove their shoes
Waist circumference	HSE 2008[a]	Waist circumference in cm was measured using a tape with an insertion buckle at one end. Two measurements were taken. A third measurement was taken if the previous two measures	Waist circumference (cm) was measured using a Wessex non-stretchable sprung tape measure.

		differed by more than 3 cm. The mean of the two measurements (or two out of three that were closest together) was derived.<br><br>Participants with a colostomy or ileostomy, or were pregnant or unable to stand were not measured	Participants were asked to adjust clothing if necessary
Smoking status	HSE 2008[a]	Verbal interview<br><br>Smoking status derived from two questions<br><br>'*May I just check, have you ever smoked a cigarette, a cigar or a pipe?*'— multiple choice response; either 1) Yes OR 2) No<br><br>'*Do you smoke cigarettes at all nowadays?*' multiple choice response; either 1) Yes OR 2) No	Touchscreen questionnaire<br><br>Smoking status was derived from two questions:<br><br>*'Do you smoke tobacco now?'*—multiple choice response; either 1) Yes, on most or all days OR 2) Only occasionally OR 3) No OR 4) Prefer not to answer.<br><br>*'In the past, how often have you smoked tobacco?'*—multiple choice response; either 1) Smoked on most or all days 2) Smoked occasionally 3) Just once or twice 4) I have never smoked 5) Prefer not to answer
Cigarette consumption	HSE 2008[a]	Verbal interview<br><br>Cigarette consumption derived from two questions.<br><br>'*About how many cigarettes a day do you usually smoke on weekdays?*'<br><br>'*About how many cigarettes a day do you usually smoke on weekends?*'	Touchscreen questionnaire<br><br>'*About how many cigarettes do you smoke on average each day?*'
Alcohol consumption	HSE 2008[a]	Verbal interview<br><br>'*Thinking now about all kinds of drinks, how often have you had an alcoholic drink of any kind during the last 12*	Touchscreen questionnaire<br><br>'*About how often do you drink alcohol?*'—multiple choice response; either 1) Daily or almost daily OR

		months?'—multiple choice response; either 1) Almost every day OR 2) Five or six days a week OR 3) Three or four days a week OR 4) Once or twice a week OR 5) Once or twice a month OR 6) Once every couple of months OR 7) Once or twice a year OR 8) Not at all in the last 12 months	2) Three or four times a week OR 3) Once or twice a week OR 4) One to three times a month OR 5) Special occasions only OR 6) Never OR 7) Prefer not to answer
Self-reported health outcomes	HSE 2006[b], 2009[c] and 2010[d]	All outcomes collected using a verbal interview<br><br>HSE 2006—CVD, IHD, stroke, angina, MI and abnormal heart rhythm<br><br>Stroke, angina, MI and abnormal heart rhythm derived from two separate questions asked for each condition individually<br><br>'*Have you ever had a stroke/angina/a heart attack (including myocardial infarction or coronary thrombosis)/an abnormal heart rhythm?'*—multiple choice response; either 1) Yes OR 2) No<br><br>'*Were you told by a doctor that you had a stroke/angina/a heart attack (including myocardial infarction or coronary thrombosis)/an abnormal heart rhythm?'*—multiple choice response; either 1) Yes OR 2) No<br><br>IHD was derived from responding yes to having a doctor diagnosed myocardial infarction or angina<br><br>CVD was derived from responding yes to having a doctor diagnosed angina, myocardial infarction, stroke, heart murmur or irregular heart rhythm<br><br>HSE 2009—hypertension and diabetes	Verbal interview by a trained nurse partially guided by touchscreen questionnaire responses.<br><br>A nurse was informed electronically whether a participant responded in the touchscreen questionnaire that they had been told by a doctor that they have one or more of the following conditions: heart attack, angina, stroke high blood pressure, blood clot in leg (DVT), blood clot in lung, emphysema/chronic bronchitis, asthma or diabetes.<br><br>The purpose of the interview was to confirm with the participant whether they had been diagnosed with any of the above health outcomes and to obtain the date of diagnosis. If, during the interview, it appeared these conditions were incorrectly selected, the responses were removed by the nurse.<br><br>If further data was required, the nurse asked the following open ended question; '*In the touchscreen you selected that you have been told by a doctor that you have other serious illnesses or disabilities, could you now tell me what they are?'*

Hypertension status derived from two questions

‘*Do you now have, or have you ever had high blood pressure (sometimes called hypertension)?*’—multiple choice response; either 1) Yes OR 2) No

‘*Were you told by a doctor or a nurse that you had high blood pressure?*’—multiple choice response; either 1) Yes OR 2) No

Diabetes status derived from two questions

Do you now have, or have you ever had diabetes?—multiple choice response; either 1) Yes OR 2) No

Were you told by a doctor that you had diabetes?—multiple choice response; either 1) Yes OR 2) No

HSE 2010—asthma and COPD

Asthma

‘*Did a doctor or nurse ever tell you that you had asthma?*’—multiple choice response; either 1) Yes OR 2) No

COPD status derived from two questions

‘*Did a doctor ever tell you that you had chronic bronchitis, emphysema or COPD (Chronic Obstructive Pulmonary Disease)?*’—multiple choice response; either 1) Yes OR 2) No

‘*Which of the following did the doctor tell you which you had?*’—multiple choice response; 1) COPD and/or 2) Chronic bronchitis and/or 3) Emphysema—for this question,

	participants were shown a card with images to help identify which condition/s
	HSE 2009 and 2010—CKD<br>CKD status derived from two questions<br>'*Do you yourself now have, or have you ever had chronic kidney disease?* '—multiple choice response; either 1) Yes OR 2) No<br>'*Were you told by a doctor that you had chronic kidney disease*'—multiple choice response; either 1) Yes OR 2) No

BMI, body mass index; CKD, chronic kidney disease; COPD, chronic obstructive pulmonary disease; CVD, cardiovascular disease; IHD, ischaemic heart disease; MI, myocardial infarction.

[a] Overall response to HSE 2008; 64% of eligible households in general population sample and 73% of eligible households in the boost sample. Individual response rate (calculated as proportion of number of individuals in sampled households) was 58% for those interviewed, 52% for those who had their height measured and 50% for those who had their weight measured. See reference 9 for further information about HSE data.

[b] Overall response to HSE 2006; 68% of eligible households in general population sample and 73% of eligible households in the boost sample. Individual response rate (calculated as proportion of number of individuals in sampled households) was 61% for those interviewed. See reference 8 for further information about HSE data.

[c] Overall response to HSE 2009; 68% of eligible households in general population sample. Individual response rate (calculated as proportion of number of individuals in sampled households) was 61% for those interviewed. See reference 10 for further information about HSE data.

[d] Overall response to HSE 2010; 66% of eligible households in general population sample. Individual response rate (calculated as proportion of number of individuals in sampled households) was 59% for those interviewed. See reference 11 for further information about HSE data.

Web Table 2. Characteristics of the Total UK Biobank Sampling Frame and Those Who Participated in the Study[a]

	UK Biobank Invitees (n = 8,761,869) No. (%)	UK Biobank Participants[b] (n = 503,310) No. (%)
**Sex**		
Men	4,468,580 (51.0)	229,486 (45.6)
Women	4,293,289 (49.0)	273,824 (54.4)
**Age group at time of invitation, years**		
40–44	1,770,821 (20.2)	53,953 (10.7)
45–49	1,780,661 (20.3)	66,438 (13.2)
50–54	1,506,431 (17.2)	76,808 (15.3)
55–59	1,366,076 (15.6)	91,953 (18.3)
60–64	1,323,219 (15.1)	121,419 (24.1)
65–70	1,014,661 (11.6)	92,739 (18.4)
**Region**		
South East	717,053 (8.2)	50,498 (10.0)
London	1,330,405 (15.2)	61,982 (12.3)
East Midlands	618,724 (7.1)	40,399 (8.0)
West Midlands	877,480 (10.0)	41,024 (8.2)
South West	440,989 (5.0)	42,340 (8.4)
North West	1,758,685 (20.1)	82,386 (16.4)
Yorkshire and the Humber	1,074,321 (12.3)	65,377 (13.0)
North East	957,005 (10.9)	61,714 (12.3)
Scotland West	435,661 (5.0)	18,576 (3.7)
Scotland East	211,357 (2.4)	17,309 (3.4)
Wales	340,189 (3.9)	21,705 (4.3)
**Townsend deprivation score[c]**		
Less deprived (<-2)	3,137,375 (35.8)	260,185 (51.8)
Average (≥-2 to <2)	2,971,606 (34.0)	159,483 (31.8)
More deprived (≥2)	2,645,024 (30.2)	82,360 (16.4)

[a] Invitees included both non-participating invitees and UK Biobank participants.

[b] Excludes 7 participants who consented but were not invited due to missing data at the invitation stage.

[c] Townsend deprivation score calculated prior to participant joining UK Biobank. Participants are assigned a score corresponding to the output area in which their postcode is located, whereby a higher score indicates increased deprivation (and the national average is zero).

Web Table 3. Comparison (%) of Body Mass Index by Age and Sex in UK Biobank Participants With Data From the Health Survey for England, 2008[a,b]

Body Mass Index	Men				Women			
	Age 45–54 years		Age 55–64 years		Age 45–54 years		Age 55–64 years	
	UK Biobank (*n*=61,860)	HSE (*n*=1,059)	UK Biobank (*n*=94,776)	HSE (*n*=968)	UK Biobank (*n*=79,714)	HSE (*n*=1,057)	UK Biobank (*n*=116,303)	HSE (*n*=985)
<18.5	0.3	0.2	0.2	0.4	0.8	0.7	0.7	0.8
≥18.5–<25	25.9	25.3	24.0	21.4	42.3	35.3	36.7	30.6
≥25–<30	48.2	43.7	49.5	44.3	33.9	28.9	38.1	37.7
≥30	25.6	31.5	26.3	35.6	23.0	32.2	24.6	34.0

[a] See reference 9 for further information about HSE data.
[b] Excludes 2,158 UK Biobank participants aged 45–64 years with missing data for BMI.

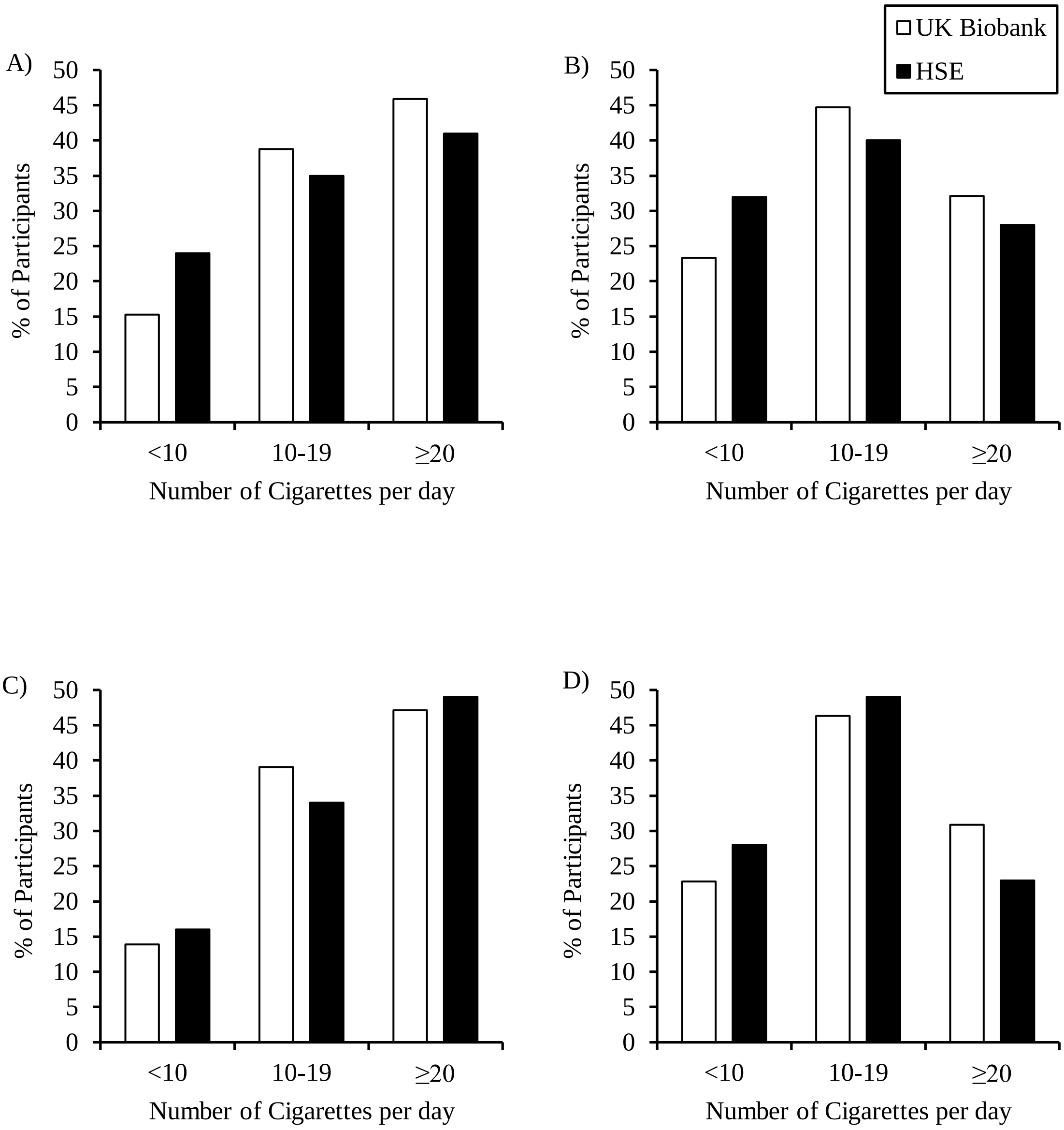

Web Figure 2. Comparison of daily number of cigarettes smoked (among smokers) in UK Biobank participants with data from the Health Survey for England (HSE), 2008 for A) men aged 45-54 years, B) women aged 45-54 years, C) men aged 55-64 years and D) women aged 55-64 years. HSE estimates are weighted for nonresponse bias. Excludes 249 UK Biobank smokers aged 45-64 years who responded ‘prefer not to answer’, ‘do not know’ or who smoked on average less than one cigarette per day. Number of participants: A) UK Biobank = 6,116, HSE = 265; B) UK Biobank = 6,628, HSE = 250; C) UK Biobank = 6,640, HSE = 192; D) UK Biobank = 7,066, HSE = 182. See reference 9 for further information about HSE data.

Web Table 4. Comparison of Mortality Rates per 1000 person-years by Age at Death for UK Biobank Participants and the Population of England and Wales in 2012 From the Office for National Statistics[a]

Age at Death, years	Men		Women	
	UK Biobank	General Population	UK Biobank	General Population
45-49	1.3	2.4	0.8	1.6
50-54	2.2	3.6	1.4	2.5
55-59	3.2	5.9	1.9	4
60-64	4.9	9.5	2.7	6.2
65-69	7.8	14.5	4.2	9.6
70-74	13.4	24.9	7.3	16.4

[a] See reference 15 for further information about death registration data.

Web Table 5. Comparison of Incidence Rates for Cancer per 100,000 Person-Years by Age at Cancer Diagnosis for UK Biobank Participants and the Population of England in 2012 from the Office for National Statistics[a]

Age at Cancer, years	Men		Women	
	UK Biobank	General Population	UK Biobank	General Population
All cancer excluding non-melanoma skin cancer				
45-49	177.7	201	438	408.2
50-54	330.5	356.9	514.7	551.2
55-59	621.6	670.7	615.8	703.4
60-64	1011.9	1,146.80	835	963.5
65-69	1644.1	1,706.30	1062.4	1,241.20
70-74	2037.5	2,310.90	1209.2	1,477.30
Prostate				
45-49	23.6	17	-	-
50-54	83.2	63.2	-	-
55-59	219.4	173.4	-	-
60-64	406.3	336.1	-	-
65-69	722.1	572.6	-	-
70-74	807.7	691.3	-	-
Breast				
45-49	-	-	280.8	222.9
50-54	-	-	274.3	271.5
55-59	-	-	260.9	263
60-64	-	-	357.3	342.7
65-69	-	-	405.4	403.2
70-74	-	-	332.5	336.2
Colon and rectum				
45-49	22.9	25.1	19.3	19.8
50-54	50.4	47.4	40	35.3
55-59	81.7	86.7	58.5	60.4
60-64	141.2	159.9	84	92.5
65-69	194.2	210.2	111.7	128.3
70-74	253.8	317.9	164.7	193.6
Lung, trachea, bronchus				
45-49	4.7	14.8	8.5	15.2
50-54	19.3	33.3	17.4	32.3
55-59	41.1	80.1	33.5	70.3
60-64	66.5	147.6	50.2	113.9
65-69	118.7	233.1	89.8	178.6
70-74	186.3	361	119.1	245.9
Endometrial				
45-49	-	-	12.5	12.1
50-54	-	-	27.8	30.3
55-59	-	-	47.4	54.3
60-64	-	-	56.8	69
65-69	-	-	70	83.5
70-74	-	-	75	91.7

Kidney				
45-49	5.4	12.2	4	6.3
50-54	14.1	18.9	5.2	9.6
55-59	25.2	26.9	10.6	14.4
60-64	32.4	42.1	17.4	21.3
65-69	48.9	52.4	20.5	21.7
70-74	54.5	65.1	26.3	35.7

[a] See reference 16 for further information about cancer registration data.

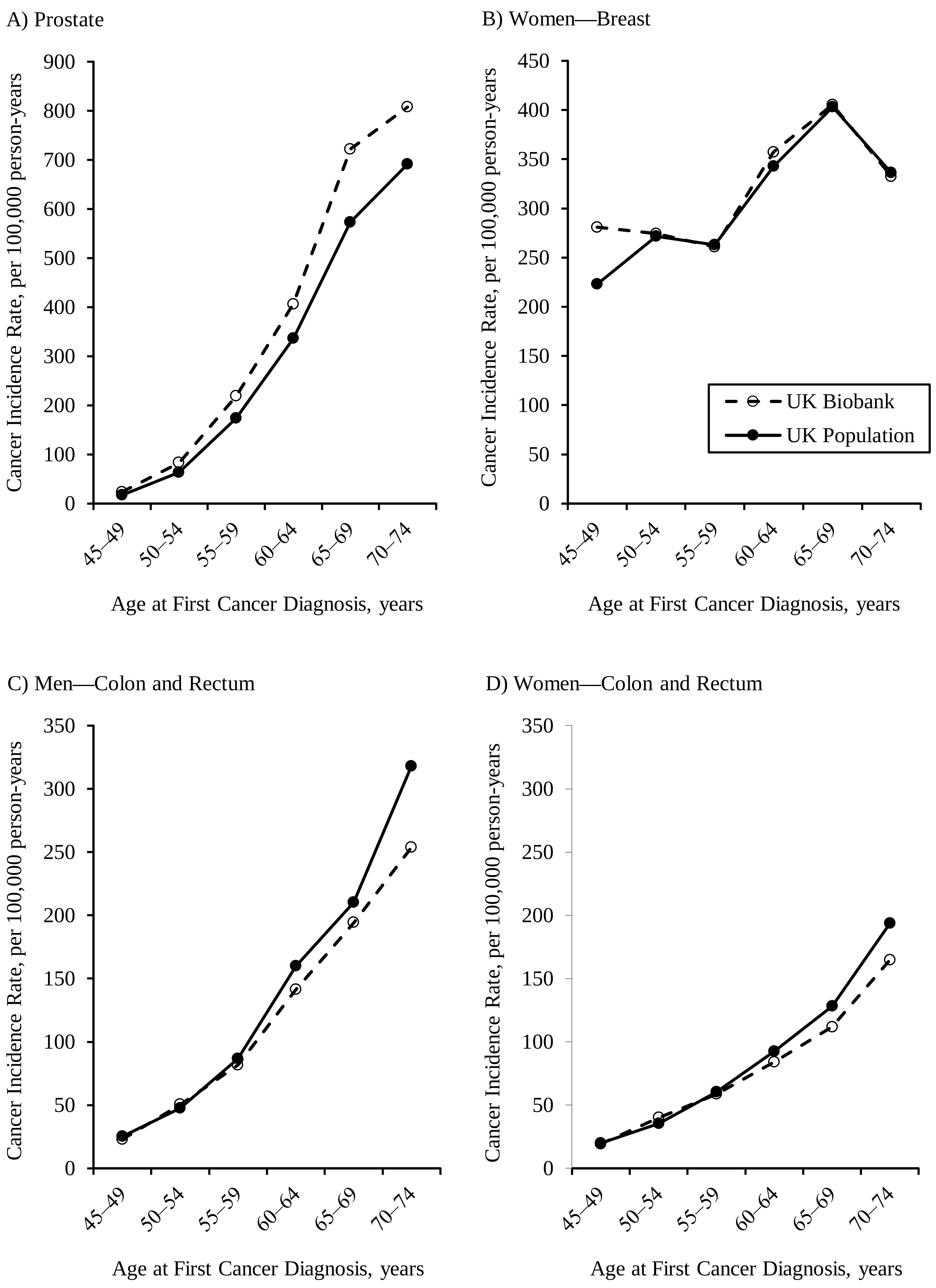
A) Prostate
B) Women—Breast
C) Men—Colon and Rectum
D) Women—Colon and Rectum
Cancer Incidence Rate, per 100,000 person-years
Age at First Cancer Diagnosis, years
0
100
200
300
400
500
600
700
800
900
0
50
100
150
200
250
300
350
400
450
45–49
50–54
55–59
60–64
65–69
70–74
UK Biobank
UK Population

E) Men—Lung, Trachea, Bronchus

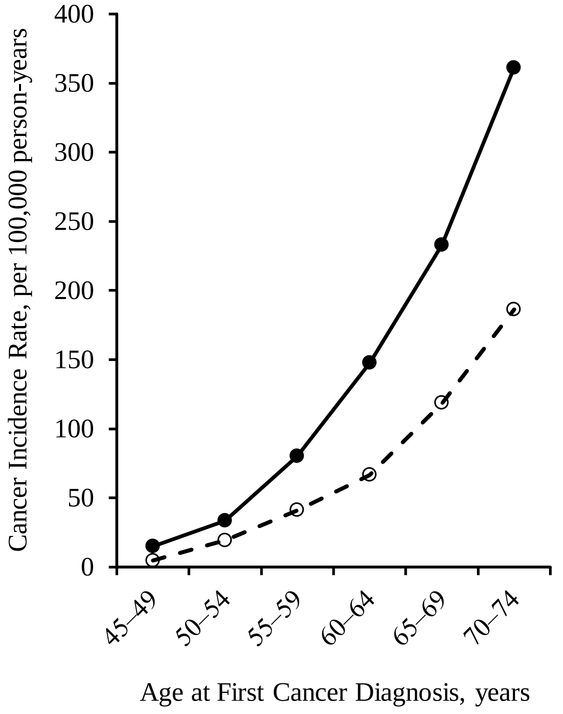

F) Women—Lung, Trachea, Bronchus

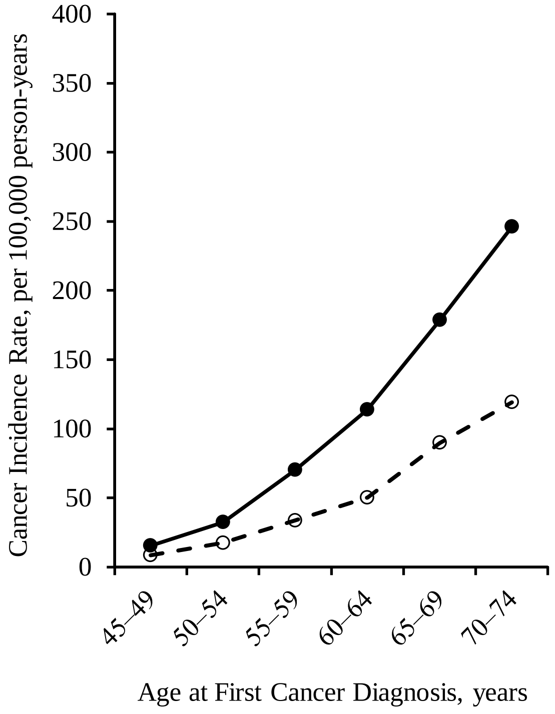

G) Endometrial

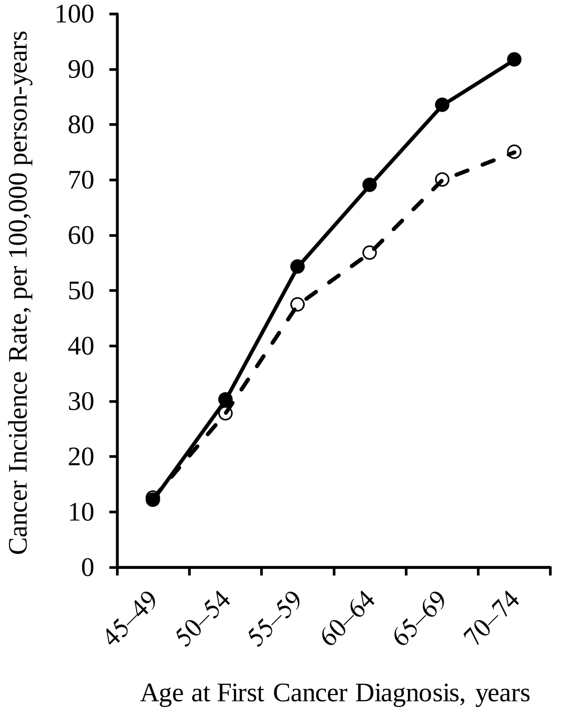

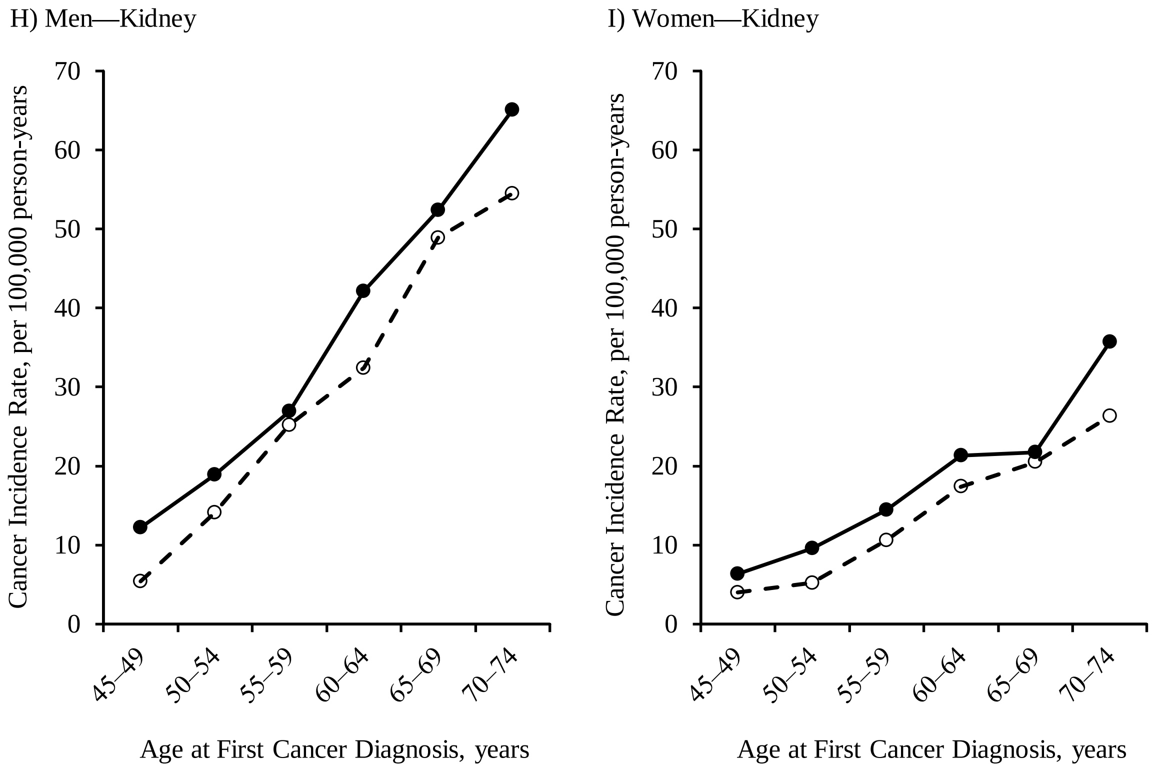

Web Figure 3. Comparison of incidence rates for A) prostate cancer, B) breast cancer, colorectal cancer for C) men and D) women, cancer of the lung, trachea and bronchus for E) men and F) women, G) endometrial cancer, kidney cancer for H) men and I) women per 100,000 person-years by age at diagnosis for UK Biobank participants and the population of England in 2012 from the Office for National Statistics. Total number of incident cancers in UK Biobank participants aged 45-74: 4,528 for prostate cancer, 4,422 for breast cancer, 1,460 (men) and 1,058 (women) for colorectal cancer, 830 (men) and 704 (women) for cancer of the lung, trachea and bronchus, 672 for endometrial cancer, 359 (men) and 191 (women) for kidney cancer. See reference 16 for further information about cancer registration data.
